# Effect of a gum‐based thickener on the safety of swallowing in patients with poststroke oropharyngeal dysphagia

**DOI:** 10.1111/nmo.13695

**Published:** 2019-08-11

**Authors:** Mireia Bolivar‐Prados, Laia Rofes, Viridiana Arreola, Sonia Guida, Weslania V. Nascimento, Alberto Martin, Natàlia Vilardell, Omar Ortega Fernández, Dina Ripken, Mirian Lansink, Pere Clavé

**Affiliations:** ^1^ Gastrointestinal Physiology Laboratory Hospital de Mataró, Universitat Autònoma de Barcelona Mataró Spain; ^2^ Centro de Investigación Biomédica en Red de Enfermedades Hepáticas y Digestivas (Ciberehd) Barcelona Spain; ^3^ Danone Nutricia Research Nutricia Advanced Medical Nutrition Utrecht The Netherlands

**Keywords:** aspiration, deglutition disorders, shear viscosity, stroke, swallow response, thickener, xanthan gum

## Abstract

**Background:**

Increasing viscosity with thickening agents is a valid therapeutic strategy for oropharyngeal dysphagia (OD). To assess the therapeutic effect of a xanthan gum‐based thickener (Nutilis Clear^®^) at six viscosities compared with thin liquid in poststroke OD (PSOD) patients.

**Methods:**

A total of 120 patients with PSOD were studied in this controlled, multiple‐dose, fixed‐order, and single‐blind study using videofluoroscopy (VFSS). A series of boluses of 10 mL thin liquid and 2000, 1400, 800, 450, 250, and 150 mPa s viscosities were given in duplicate, interrupted in case of aspiration. We assessed the safety and efficacy of swallow and the kinematics of the swallow response.

**Key Results:**

A total of 41.2% patients had safe swallow at thin liquid which significantly increased for all viscosities from 71.9% at 150 mPa s to 95.6% at 1400 mPa s (*P* < .001). PAS score (3.7 ± 2.3) at thin liquid was also reduced by increasing bolus viscosity (*P* < .001). The prevalence of patients with aspiration at thin liquid was 17.5% and decreased at all viscosities (*P* < .01), except at 150 mPa s. Increasing viscosity shortened time to laryngeal vestibule closure (LVC) at all viscosities (*P* < .01) and reduced bolus velocity at ≥450 mPa s (*P* < .05). The prevalence of patients with pharyngeal residue at each viscosity 37.7%‐44.7% was similar to that at thin liquid (41.2%).

**Conclusions and Inferences:**

The prevalence of unsafe swallow with thin liquids is very high in PSOD. Increasing shear bolus viscosity with this xanthan gum‐based thickener significantly increased the safety of swallow in patients with PSOD in a viscosity‐dependent manner without increasing the prevalence of pharyngeal residue.

## Graphical abstract

This study shows a strong therapeutic effect of a gum‐based thickener on safety of swallow in post‐stroke dysphagia that depends on shear viscosity levels, and a therapeutic range of 150‐800 mPa s. Increasing viscosity shortened time to laryngeal vestibule closure at all viscosities and reduced bolus velocity at ≥450 mPa s.

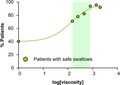

**Key Points**

- Oropharyngeal dysphagia (OD) occurs in 45% poststroke patients. Increasing bolus viscosity with thickeners reduces aspirations, but optimal viscosity levels need to be determined.
- We assessed 7 shear viscosity levels with a xanthan gum‐based thickener in stroke patients with dysphagia and found a viscosity‐dependent improvement in swallowing safety from 150 mPa s to 800 mPa s through reduced time to laryngeal vestibule closure and bolus velocity.
- This is the first study to show the full dynamics and mechanisms of gum‐based thickeners in poststroke OD.

## 1 INTRODUCTION

Oropharyngeal dysphagia (OD) is a motility disorder characterized by difficulty forming or moving the alimentary bolus from the mouth to the esophagus and can include aspiration.^1^ Poststroke OD (PSOD) is classified in the ICD under the code: 438.82 (ICD‐9) and I69.391 (ICD‐10).^2^ OD is a prevalent complaint following stroke, with high incidence (45%) on hospital admission.^3^ It is associated with poor short‐ and long‐term prognosis and several complications, such as malnutrition, dehydration,^4^ and aspiration pneumonia, increasing the risk of mortality 5, 6, 7 in comparison with poststroke patients without OD.^5^, ^8^, ^9^, ^10^ It is an independent risk factor for prolonged hospital stay and institutionalization after discharge, and for poorer functional capacity and increased mortality 3 months after stroke.^3^ While some patients recover spontaneously, 50% assessed 6 months poststroke were found to have chronic OD.^11^ The pathophysiology of PSOD is characterized by several motor impairments in the kinematics of the swallow response including delayed laryngeal vestibule closure (LVC) and decreased bolus propulsion forces 12; also, patients affected by unilateral stroke showed a disrupted pattern of sensory cortical activation after pharyngeal stimulation as a distinctive marker of abnormal sensory integration of swallowing pathways in PSOD.^13^

Thickening agents increase the viscosity of fluids and thin liquids, enhancing the safety of swallow by avoiding aspirations and their associated complications,^14^, ^15^ as stated in a review by the European Society for Swallowing Disorders (ESSD).^16^ Viscosity is a rheological property which measures the resistance of a fluid to flow, expressed in SI units as mPa s 15, 17—rheology is the study of the flow and deformation of fluids.^18^, ^19^ Several factors can affect the viscosity of thickened fluids: salivary α‐amylase breaks down starch molecules during the oral phase of swallow,^15^ and shear thinning decreases viscosity with increasing bolus velocity and shear rate 18, 20 in the pharyngeal phase.

The ESSD review also recommended (a) the development of new thickening agents with less residue, more palatability and, thus, better compliance (gum‐based thickeners have proven to be better than starch) 20; and (b) clinical trials to establish the optimal viscosity level for each phenotype of dysphagic patients.^16^ Few viscosity levels per product have been studied, and the optimal viscosity levels for patients suffering poststroke OD have not been determined yet.^16^

The aim of this study was to assess the effect of a gum‐based thickener (Nutilis Clear^®^) on the safety and efficacy of swallowing in patients with poststroke OD by evaluating seven different shear viscosities (150‐2000 mPa s) during swallowing with videofluoroscopy swallowing study (VFSS). There are no previous studies that have evaluated such a wide range of viscosities. The primary objective was to assess the percentage of patients that could swallow safely at each of the three main viscosities (2000, 800 or 250 mPa s) compared with thin liquid. Secondary and exploratory objectives were to assess the effect of all viscosities on penetrations, aspirations, the Penetration‐Aspiration Scale (PAS) developed by Rosenbek,^21^ the presence and severity of oral and pharyngeal residue, and the effects on the biomechanics of the swallow response.^13^, ^15^

## 2 PATIENTS AND METHODS

### 2.1 Study population

This study included 120 PSOD outpatients who were consecutively recruited from March 2016 to December 2017 at the GI Physiology Lab of the Hospital de Mataró, Barcelona following hospital discharge. Assuming discordant proportions of 7.5% (safe swallow on thin liquid and unsafe swallow on main viscosities) and 30% (unsafe swallow on thin liquid and safe swallow on main viscosities), a sample size of 95 patients would be sufficient to have 90% power to detect statistical significant differences in safe swallowing between each of the three main viscosities and thin liquid, using a two‐sided Mcnemar's test with an α of 0.5, assuming 20% of patients do not complete the measurements. Main inclusion criteria were patients older than 18 years, minimum of 28 days since diagnosis of stroke, clinical signs or symptoms of swallowing dysfunction in the volume‐viscosity swallow test (V‐VST) 22 or referral by physician for VFSS or current use of thickened products, no alteration in consciousness, and written informed consent. Main exclusion criteria were need of oxygen therapy, OD not related to stroke, history of other neurological disorders or head and neck cancer, xerostomia induced by drugs, severe cognitive disorder, incapability to perform VFSS, pregnancy or lactation, participation in another research study, and allergy to any ingredient tested. In addition, for the description of study population, we collected demographic parameters such as age, sex, weight, height, type of stroke, time after stroke, severity of dysphagia, nutritional status, comorbidities, medication, and stroke severity according to the National Institute of Health Stroke Scale (NIHSS).^23^

The Ethics Committee of the Hospital de Mataró (Spain) approved the study protocol, information given to patients about the study and the informed consent form with code 41/15. The study was conducted according to the principles of the “World Medical Association Declaration of Helsinki” (2013) and the International Conference on Harmonization (ICH) guidelines for Good Clinical Practice (GCP, September 1997) as appropriate for nutritional products legislation of Spain where the study took place. This study has been registered in The Netherlands Trial register with code: NTR5628.

### 2.2 Experimental design

**Figure 1:**
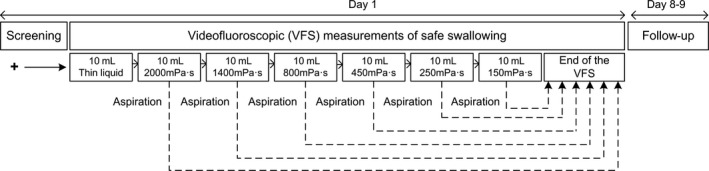
Study design

This was a reference‐controlled, multiple‐dose, fixed‐order, single‐blind, and single‐center study. The study procedure (Figure 1) was performed in one single visit. Firstly, the V‐VST—a clinical assessment tool for dysphagia—was performed on each patient to assess clinical signs of OD 23, 24, 25 and those positive for OD were referred for VFSS. One week after the completion of the study, a follow‐up call was performed to assess potential adverse events.

During the VFSS, 10mL boluses were given in duplicate to each patient, following the algorithm shown in Figure 1 (only one bolus is shown in the algorithm, but two were given if the patient swallowed safely). Briefly, the procedure started with thin liquid (when aspirations occurred, the second bolus of thin liquid was not administered to protect patients from a new aspiration) and continued with boluses from the highest viscosity to the lowest. If the patient aspirated any of the thickened boluses, the study was terminated to avoid any further aspiration as a safety measure.^16^, ^26^

### 2.3 Outcome parameters

The main outcome parameter was the percentage of patients with safe swallow (PAS score 1 and 2) 21 for the main viscosities (250, 800, and 2000 mPa s). Secondary outcome parameters were as follows: (a) safety of swallowing expressed by the mean PAS score,^21^ and the percentage of patients with penetration (PAS score of 3,4,5), or aspiration (PAS score of 6,7,8); and (b) the efficacy of swallowing expressed by the presence and severity of oral and pharyngeal residue. Exploratory parameters included physiology of swallowing (time to LVC, total duration of swallowing response –LVO–, mean bolus velocity, and bolus propulsion force), distribution of PAS scores, subjective swallowing experience at all viscosities (150, 250, 450, 800, 1400, and 2000 mPa s) and safety and efficacy of swallowing at the 3 exploratory viscosities (150, 450, and 1400 mPa s). Due to the relevance of the information for patient safety, comparisons on the prevalence of patients with safe swallow and mean PAS scores were also performed between all the different viscosities assayed in this study.

### 2.4 Methods

#### 2.4.1 Videofluoroscopy (VFSS)

VFSS is a dynamic radiological exploration that evaluates the swallowing process of boluses of various volumes and viscosities marked with a radiopaque iodine contrast.^12^ Boluses were tested while the patient was seated in a lateral projection. Boluses were prepared with water, X‐ray contrast solution (Omnipaque^™^, GE Healthcare), and the required amount of thickener (g) to achieve each viscosity level (mPa s). In our research group, volumes of 5, 10, and 20mL are routinely used in the clinical practice to test the swallowing ability of the patient in an effort test.^12^, ^22^ As not all the patients are capable of swallowing the maximum volume (20mL), 10mL was chosen as an optimal comfortable bolus for the patient to swallow in this study. The oral cavity, pharynx, larynx, and cervical esophagus were recorded on video during swallowing. VFSS recordings were obtained using a Super XT‐20 Toshiba Intensifier (Toshiba Medical Systems Europe) and recorded at 25 frames/s using a Canon DM‐XM2 E video camera (Canon Inc.). The VFSS recordings were analyzed and the measurements obtained using specialized software (Swallowing Observer; Image & Physiology SL) by an expert blinded clinician.^14^ VFSS signs. Safety of swallow was assessed by the identification of the PAS score and the prevalence of safe swallows (PAS 1,2), penetrations (PAS 3,4,5), or aspirations (PAS 6,7,8) of each bolus.^21^ We considered the following signs as indication of impaired efficacy: piecemeal deglutition, oral, pharyngeal wall, and vallecular or pyriform sinus residue. The prevalence of residue was described as the presence or absence of residue in the oral cavity or the pharynx including the pharyngeal wall, the vallecula, and pyriform sinus.^27^ Timing of oropharyngeal swallow response (OSR) and bolus kinematics. Timing of swallow response was assessed for each bolus given to the patient during VFSS.^4^, ^27^ We measured the time to LVC (time from the glossopalatal junction (GPJ) opening to the LVC) and the total duration of the swallow response. In addition, mean bolus velocity of the bolus between the GPJ and the upper esophageal sphincter (UES), propulsion forces, and kinetic energy were calculated as published before by our group.^4^

#### 2.4.2 Bolus rheology

The viscosity levels used in the study were selected according to the descriptors of the National Dysphagia Diet Task Force: 1‐50 mPa s for thin liquid, 51‐350 mPa s for nectar, 351‐1750 mPa s for honey, and >1750 mPa s for pudding viscosities at 25°C and 50 seconds^−1^.^28^ For VFSS, we prepared a total of seven different viscosities in 10 mL bolus, consisting of liquid X‐ray contrast as control vs six thickened X‐ray contrasts thickened with Nutilis Clear^®^—consisting of maltodextrin, xanthan gum, and guar gum (Nutricia N.V., Zoetermeer, The Netherlands)—at each viscosity level. To achieve those viscosities, varying amounts of thickener were added to 50 mL solution composed of 1:1 mineral water and the iodine X‐ray contrast: 150 and 250 mPa s viscosities were obtained by adding 0.56 g and 0.75 g, respectively; 450, 800, and 1400 mPa s viscosities were obtained by adding 1.27 g, 2.08 g, and 3.81 g, respectively; and 2000 mPa s was obtained with 5.01 g.

#### 2.4.3 Comfortability

During the VFSS, patients were asked whether they felt comfortable during the swallowing experience (“*I felt comfortable during swallowing this product*”) using a 9‐point Likert scale, at each viscosity level. Results are presented by using three categories: (a) strongly agree, agree, and moderately agree; (b) mildly agree, undecided, and mildly disagree; and (c) moderately disagree, disagree, and strongly disagree.

#### 2.4.4 Safety of the product

All adverse events (AEs) occurring during the study and one week after the procedure (follow‐up telephone call) were recorded and assessed for relationship with the study product according to the guideline of categories described by the World Health Organization and the Uppsala Monitoring Centre (WHO–UMC).^29^

#### 2.4.5 Data analysis and statistical methods

Binary data were described as relative and absolute frequencies, and the viscosity levels were compared with thin liquid by applying the McNemar's test. For ordinal data, the comparisons were done by applying the Bhapkar's test; in case of zero counts, the McNemar's test on aggregated categories was used. Continuous data are presented as mean ± standard deviation (SD), and comparisons were done by applying a repeated measure mixed model including all six viscosities or a paired‐sample Wilcoxon signed rank test in case assumptions were not met. The McNemar's test, Bhapkar's test, paired‐sample Wilcoxon signed rank test, and repeated mixed model all take into account the within‐subject design and paired data. The statistical analysis was performed with SAS^®^ software for Windows, SAS Institute Inc. (SAS version 9.4_M1).

Safety of swallow of each patient at a particular viscosity level was expressed as the worst PAS score of the duplicates, and all the parameters of that replicate were analyzed according to the scheme in Figure S1. Data on safety of swallowing were handled as binary by dividing the patients in two categories: patients who can swallow safely (PAS 1‐2) vs patients who cannot swallow safely (PAS 3‐8) over the “per protocol” population. The efficacy of swallowing was also handled as binary data (presence or absence): if residue was observed at any of the three pharyngeal locations (pharyngeal wall, vallecular, and pyriform sinus), the residue was present (yes); if no residue was observed at any of the locations, the residue was absent (no); and if at least one was missing (not performed due to the safety rule) and the others were absent, the residue was handled as missing. Data of the duplicates for residue were handled according to the algorithm shown in Figure S1. For efficacy of swallow, an additional procedure for handling duplicates was used to explore the “worst case” scenario. This selection was independent of PAS score, and the replicate was selected based on the worst value for the presence of pharyngeal or oral residue.

Statistical tests were conducted two‐sided with a significance level of 5%. All confidence intervals are presented two‐sided with a confidence level of 95%. A resultant probability value of *P* < .05 was judged as statistically significant. For the primary outcome parameter, percentage of patients that swallow safely, the null‐hypothesis of no effect on safe swallowing of 2000, 800, and 250 mPa s compared with liquid will be rejected if all three (two‐sided) *P* values are <.05 with correct directional decisions. An additional explorative analysis was performed on safety of swallowing and the mean PAS scores to evaluate the therapeutic effects between viscosities. As a post hoc test, the bolus propulsion force was analyzed, and dose‐response curves for the viscosity‐dependent effect of the thickening agent on safety and efficacy were obtained by representing the prevalence of patients with safe swallowing and those with residue respectively at each level of viscosity using Graphpad Prism 6.

## 3 RESULTS

### 3.1 Sample demographics

Of the 120 patients enrolled, 4 were excluded from the all subjects treated (AST) population because they did not receive any of the thickened viscosities. Additionally, two patients were excluded from the per protocol population (PP) because they discontinued due to reasons other than aspiration which was regarded as a protocol deviation. The originally planned analysis was on the intention‐to‐treat population (ITT). However, because there were 4 patients in this population who did not receive any of the thickened product, it was decided to present the results for the PP population (n = 114) (Figure S2). The results of the ITT and PP populations were comparable. The majority of our population, 76% (N = 87) were in the subacute phase (28‐180 days after stroke) and 24% (N = 27) were chronic (>180 days after stroke). Mean age of the participants was 76.7 ± 8.9 years, and 54.4% were men. The MNA‐SF total score indicated that 54.4% of patients were malnourished or at risk of malnutrition when enrolled in the study. Stroke type was predominantly ischemic 78.1% (n = 89), and the prevalent severity of the stroke valued with the NIHSS was scored (mean ± SD) 7.5 ± 6.8 on admission and 5.3 ± 5.9 on discharge. More details of the epidemiological and clinical characteristics of the population are provided in Table S1.

### 3.2 Effect of range of viscosities on prevalence of VFSS signs of OD

#### 3.2.1 Safety of swallow

##### Primary parameter

**Figure 2:**
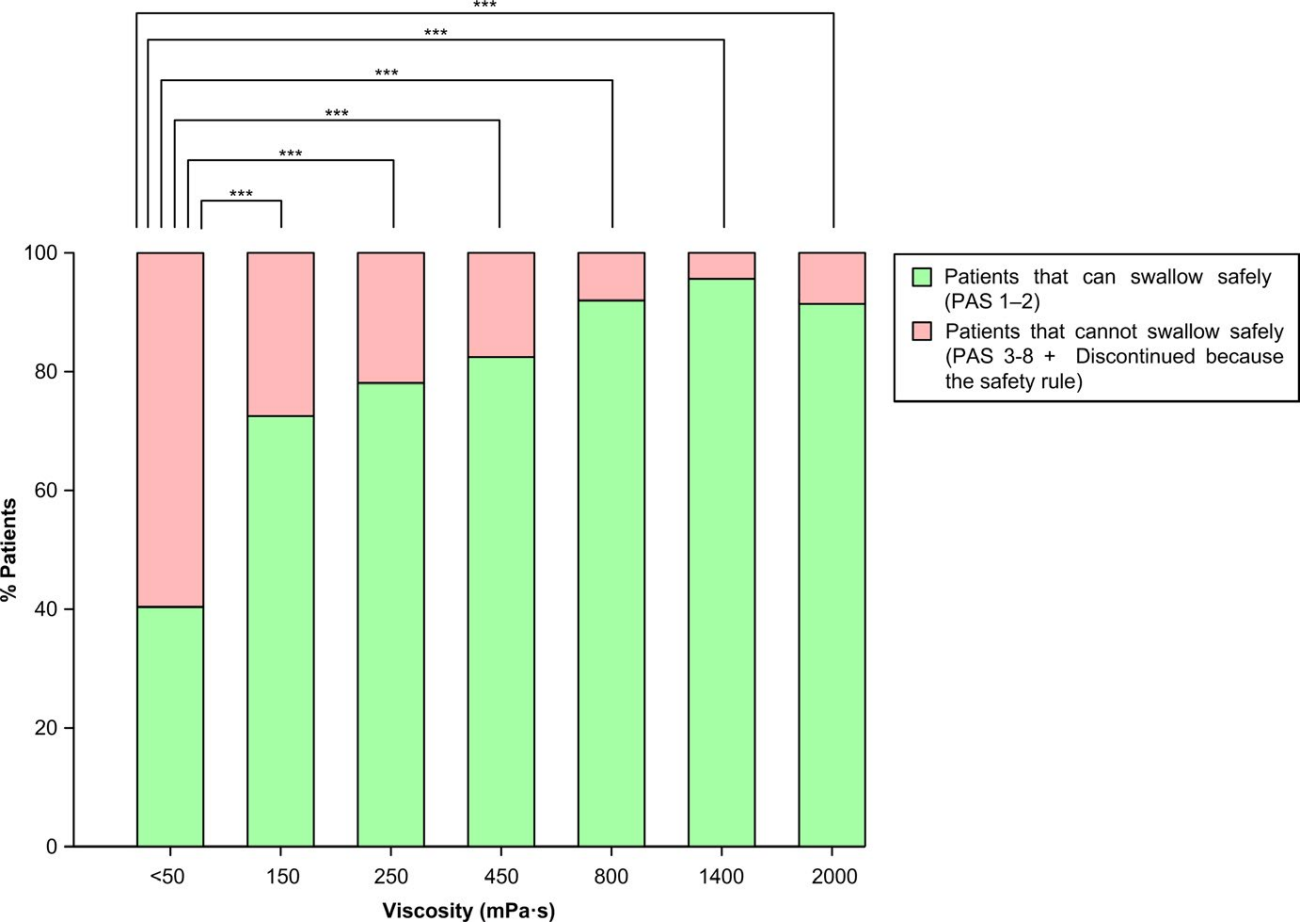
Percentage of PSOD patients with safe/unsafe swallow at each level of viscosity. “N” represents the number of patients who performed the bolus out of the PP population (114). The percentage of patients with unsafe swallow includes those with aspirations at the former viscosity who discontinued due to the safety rule. Percentage of patients who discontinued at each viscosity: thin liquid (0.0%), 150 mPa s (12.3%), 250 mPa s (8.8%), 450 mPa s (4.4%), 800 mPa s (1.8%), 1400 (1.8%), 2000 mPa s (0.9%). *P < .05; **P < .01; ***P < .001 vs thin liquid

Safe swallowing was observed in only 41.2% (n = 47) of the patients at thin liquid but the percentage significantly increased with the main viscosities (all *P* < .001 vs thin liquid) (Figure 2). Similarly, safety of swallowing significantly increased with the explorative viscosities compared with thin liquid (all *P* < .001 vs thin liquid) (Figure 2).

Mean PAS score at thin liquid was 3.7 ± 2.3, and it significantly decreased to 1.9 ± 1.4, 1.8 ± 1.6, 1.7 ± 1.6, 1.4 ± 1.2, 1.2 ± 0.6, and 1.4 ± 1.2 by increasing viscosity from 150 to 2000 mPa s (all *P* < .001 vs thin liquid). The distribution of safe swallowing, penetration, and aspiration was significantly different at all viscosities compared with thin liquid (all *P* < .001 vs thin liquid). The percentage of patients with penetration and aspiration decreased when viscosity increased (Figure S3). The prevalence of patients with penetrations at thin liquid was 41.2% and ranged between 2.6% and 13.2% for the thickened viscosities. The prevalence of patients with aspirations showed significant differences (*P* < .01) with thin liquid (17.5%) vs all viscosities (0.0%‐4.4%) except for 150 mPa s (2.5%, *P* = .180).

**Figure 3:**
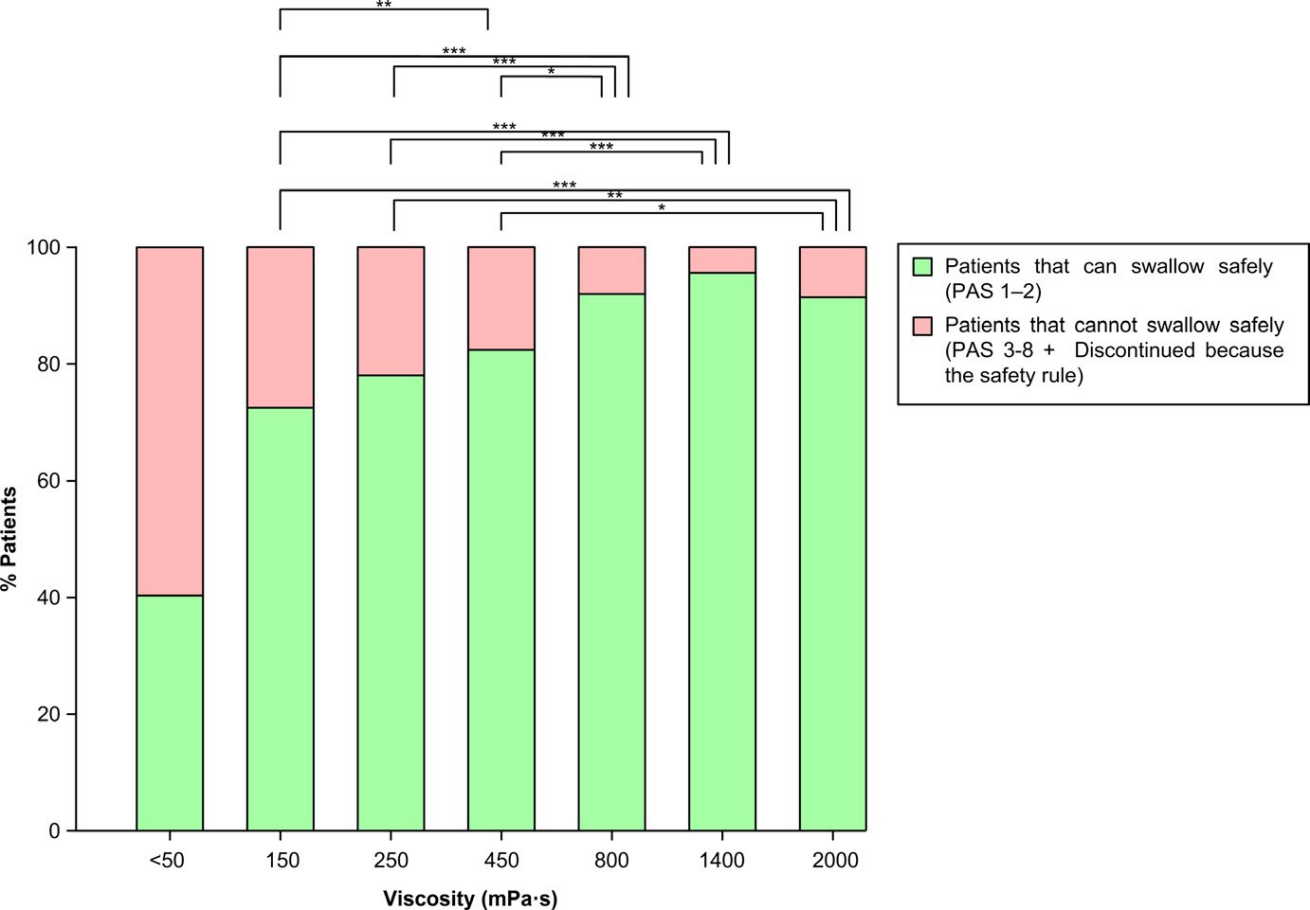
Percentage of PSOD patients with safe/unsafe swallow compared between levels of viscosity. Data of patients who discontinued due to the safety rule were imputed with the last observation carried forward. Values are presented for the PP population (114).*P < .05; **P < .01; ***P < .001

Figure 3 shows the explorative analysis of the between viscosity comparisons. Among the different viscosity levels, there were significant differences between the therapeutic effect of 250 mPa s (78.9%) vs 800 (92.1%), 1400 (95.6%), and 2000 mPa s (91.2%) (all *P* < .01 vs 250 mPa s), but not between 800 and 2000 mPa s or between 800 and 1400 (*P* > .05). The maximal therapeutic effect (ceiling effect) was observed at 800 mPa s (92.1% of patients with safe swallowing).

#### 3.2.2 Efficacy of swallow

**Figure 4:**
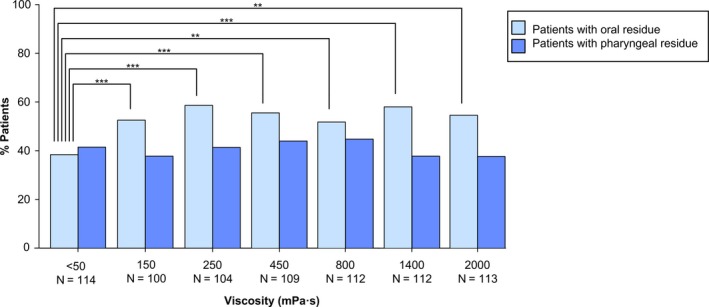
Percentage of patients with PSOD of the PP population (114) with oral and pharyngeal residue at each viscosity level. “N” represents the population who performed the bolus. *P < .05; **P < .01; ***P < .001 vs thin liquid

At thin liquid, pharyngeal residue was present in 41.2% (n = 47) of patients and it did not increase at any of the tested viscosities (37.7%‐44.7%, all *P* > .05 vs thin liquid) (Figure 4). Oral residue was present in 38.6% (n = 44) at thin liquid and significantly increased at all thickened viscosities (all *P* < .01 vs thin liquid) (Figure 4). Selecting the duplicate with the “worst case” scenario resulted in comparable results (not shown).

#### 3.2.3 Dose‐response effect of range of viscosities on safety of swallowing and pharyngeal and oral residue

**Figure 5:**
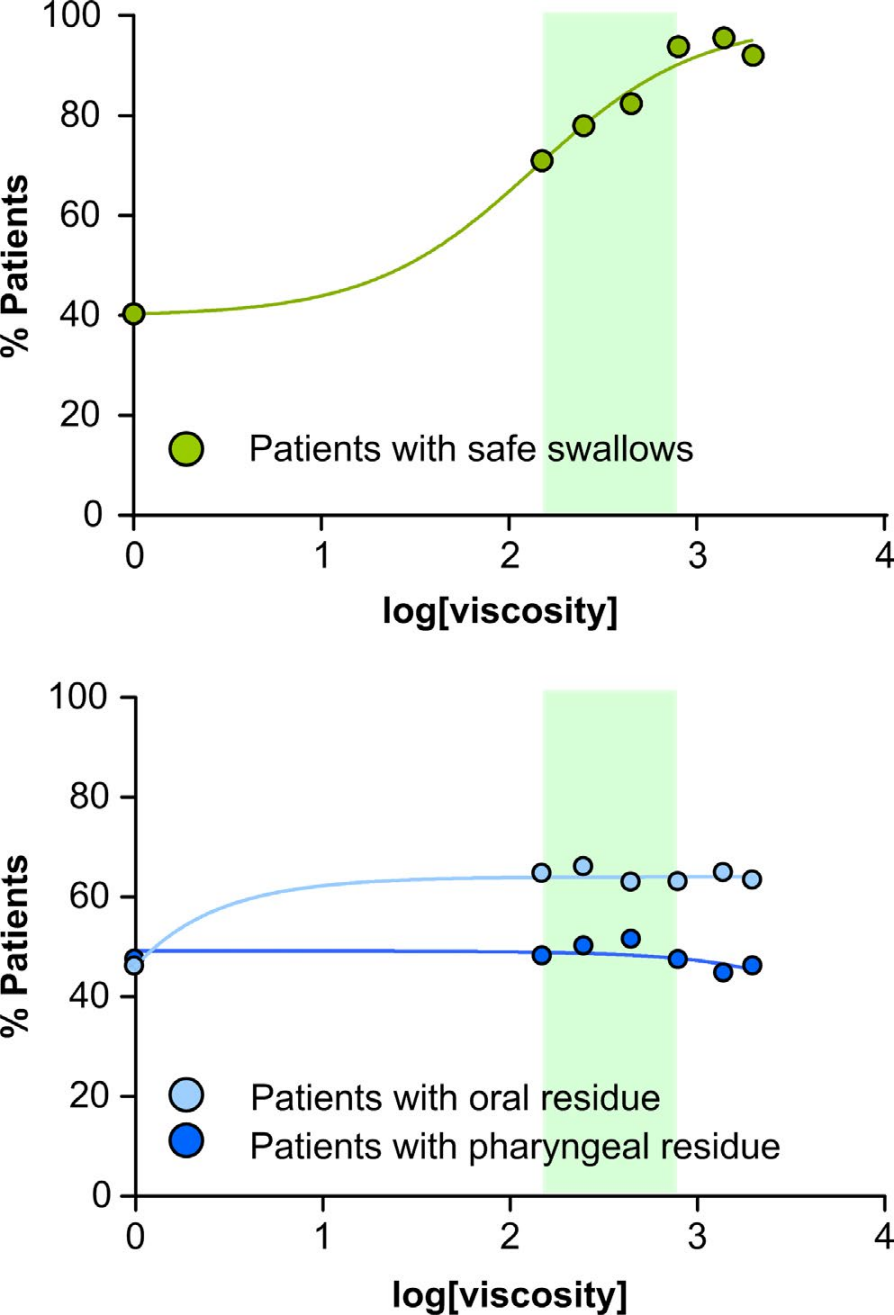
Dose‐response curves for the therapeutic effect of the gum‐based thickener on safety and efficacy of swallowing in patients with PSOD. The upper panel shows the curve of the viscosity‐dependent response represented by the percentage of patients with safe swallows vs the log of the viscosity. The lower panel shows the curve representing the effects on the prevalence of oral and pharyngeal residue vs the log of the viscosity. The shadowed area represents the therapeutic range (150‐800 mPa s) of the product

Figure 5 shows the viscosity‐dependent therapeutic effect on safety of swallowing for the tested viscosities. 150, 250, and 450 mPa s offered a protection on safety of swallowing between 71.9% and 82.5% and 800, 1400, and 2000 mPa s a protection between 91.2% and 95.6%. Safety increased in a viscosity‐dependent manner. Pharyngeal residue was not statistically different compared with thin liquid at any of the tested viscosities. Oral residue slightly, but significantly, increased at all viscosities.

### 3.3 Effect of range of viscosities on oropharyngeal swallow response (OSR)

#### 3.3.1 Timing of OSR

##### Time to laryngeal vestibule closure (LVC)

**Figure 6:**
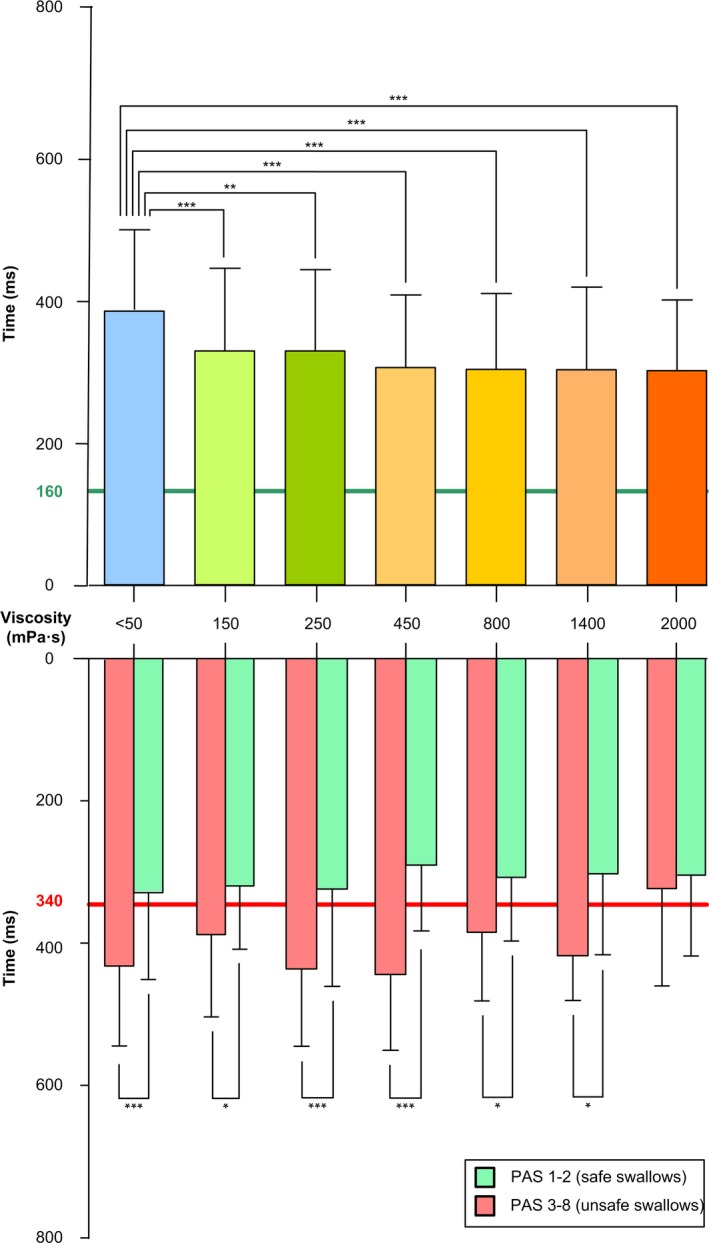
Time to LVC at each viscosity level. The upper panel shows mean time to LVC at each viscosity. The lower panel shows time to LVC plotted against safe/unsafe swallow at each viscosity level. Time to LVC was delayed in patients with unsafe swallowing at all viscosity levels except for 2000 mPa s. Time to LVC <160 ms (green line): safe swallowing as established in a study with healthy volunteers.^4^Time to LVC ≥340 ms (red line): cutoff time to detect the presence of unsafe swallowing in poststroke patients according to previous studies.^12^ *P < .05; **P < .01; ***P < .001

Time to LVC at liquid viscosity was severely delayed (382.5 ± 139.1 ms) in patients with PSOD. Increasing bolus viscosity ≥150 mPa s shortened time to LVC for all viscosities (Figure 6): mean LVC for each viscosity was 327.3 ± 108.2 (150 mPa s), 330.1 ± 143.4 (250 mPa s), 304.8 ± 109.6 (450 mPa s), 303.3 ± 94.7 (800 mPa s), 300.5 ± 110 (1400 mPa s), and 300.4 ± 107.8 (2000 mPa s) ms (*P* < .01 vs liquid). Time to LVC was shorter in patients with safe (PAS 1‐2) vs unsafe swallow (PAS 3‐8): significant differences were detected in all viscosities except for 2000 mPa s (Figure 6).

At thin liquid, the total duration of the swallow response was 1020.9 ± 220.8 ms and significantly decreased to 947.1 ± 228.7, 998.8 ± 472.1, 944.1 ± 180.2, 943.1 ± 221.4, 953.5 ± 225.3, and 943.2 ± 234.8 ms at 150, 250, 450, 800, 1400, and 2000 mPa s, respectively (all *P* < .01 vs thin liquid).

#### 3.3.2 Bolus kinematics

##### Mean bolus velocity

**Figure 7:**
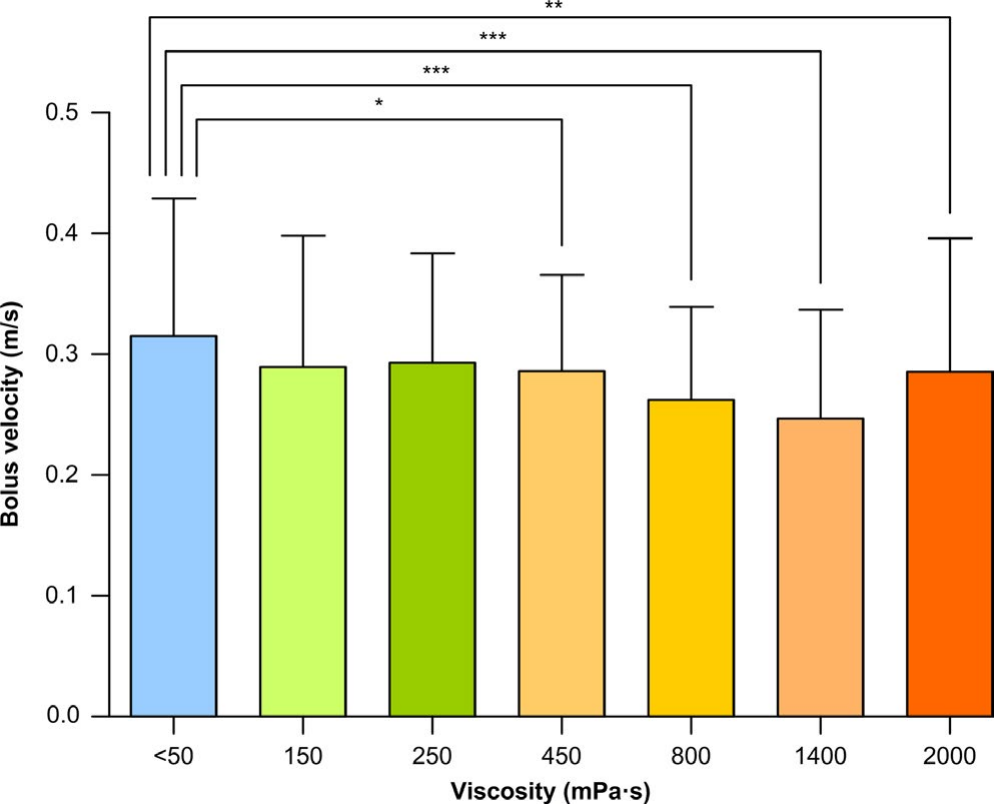
Mean bolus velocity from GPJO to UESO at each viscosity level. Bolus velocity was reduced above 450 mPa s. *P < .05; **P < .01; ***P < .001 vs thin liquid

Poststroke patients included in the study presented a mean bolus velocity at liquid of 0.3138 ± 0.1265 (m/s). Increasing bolus viscosity, ≥450 mPa s, caused a significant reduction in bolus velocity for 450 mPa s (0.2835 ± 0.0948; *P* < .05), 800 mPa s (0.2613 ± 0.0784; *P* < .001), 1400 mPa s (0.2564 ± 0.0803; *P* < .001), and 2000 mPa s (0.2729 ± 0.1010; *P* < .01) vs thin liquid (Figure 7).

##### Bolus propulsion forces

Mean bolus propulsion force was 0.041 ± 0.035 mN at thin liquid. A significant decrease was found at the thickened viscosities (all *P* < .001 vs thin liquid): 150 mPa s (0.033 ± 0.025), 250 mPa s (0.035 ± 0.032), 450 mPa s (0.030 ± 0.019), 800 mPa s (0.026 ± 0.014), 1400 mPa s (0.025 ± 0.015), and 2000 mPa s (0.028 ± 0.022).

### 3.4 Comfortability

**Figure 8:**
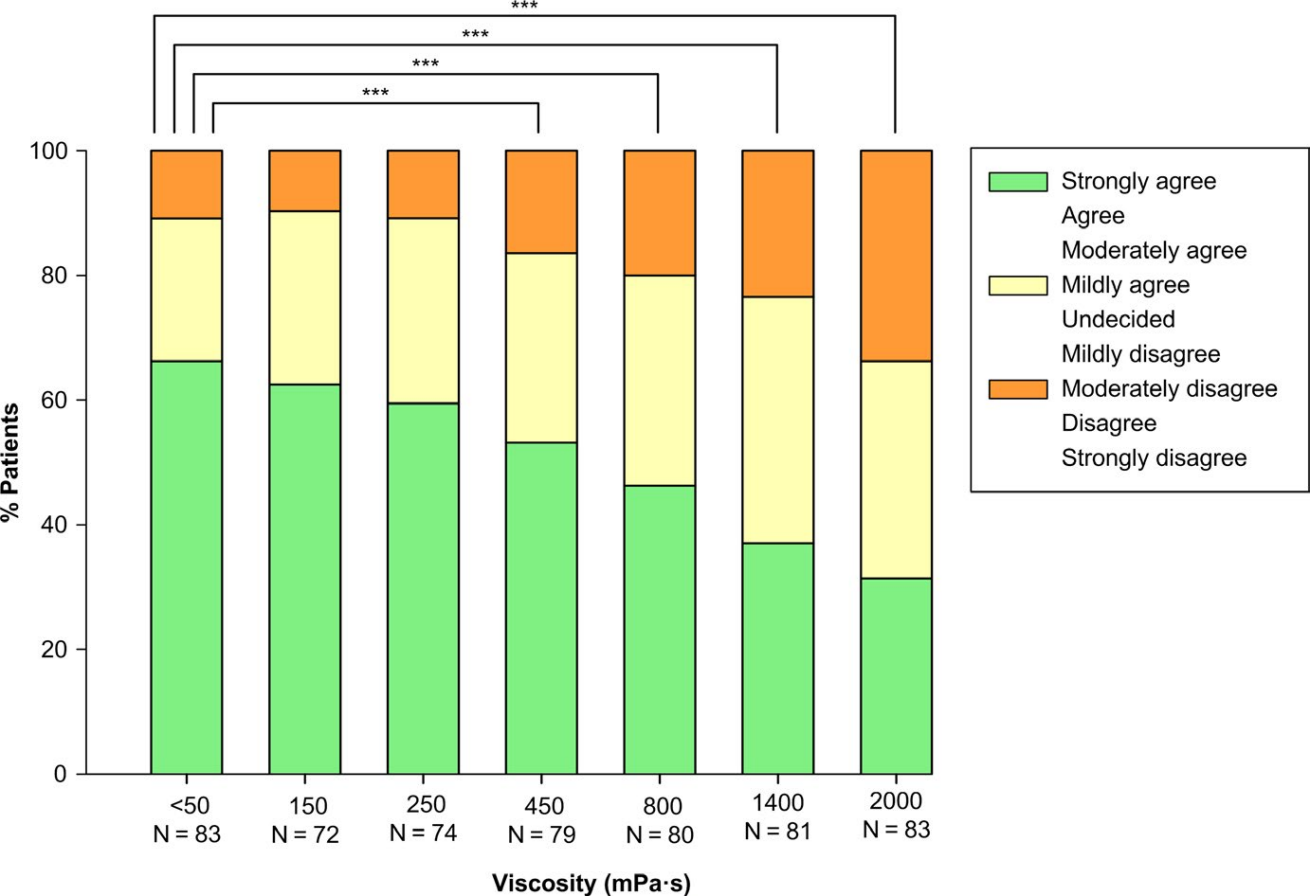
Comfortability while swallowing the product. The comfortability while swallowing the product at each viscosity level was evaluated by using a 9‐point Likert scale to the following sentence: “I felt comfortable while swallowing this product.” Likert scale score is divided into three categories for each viscosity. For the statistical analysis, these three catergories and the category of missing values were used. “N” represents the population who answered the question, the category of missing values is not shown in the figure. *P < .05; **P < .01; ***P < .001 vs thin liquid

Comfortability while swallowing scored highest at thin liquid (66.3%), and it decreased significantly to 46.3% and 31.3% during swallowing the main viscosities 800 and 2000 mPa s, respectively (Figure 8). Categories of comfortability were differently distributed at all viscosities compared with thin liquid (all *P* < .001 vs thin liquid), except for 150 and 250 mPa s (Figure 8).

### 3.5 Safety of the product. Adverse events (AEs)

A total of 16 adverse events occurred in 11 patients out of the 116 in the AST population and were considered unrelated or unlikely to be related to the study product. The most frequent AEs were mild gastrointestinal disorders (14 AEs in 10 patients): diarrhea, nausea, abdominal distension and pain, dyspepsia, and stomatitis. No serious AEs were reported during or following the study.

## 4 DISCUSSION

The main result of this study is that increasing bolus viscosity with the xanthan gum‐based thickener Nutilis Clear^®^ significantly increased the safety of swallow in patients with PSOD in a viscosity‐dependent manner. The study also shows that these patients presented a pattern of OD with highly prevalent and severe signs of impaired safety and efficacy of swallow, aspirations and oropharyngeal residue, a severely impaired swallow response, and a high prevalence of malnutrition or risk of malnutrition. Together these characteristics place these patients at high risk for severe nutritional and respiratory complications. Impaired safety of swallow in these patients with PSOD is associated with a severe delay in time to LVC. An unexpected, but very relevant, result of this study was that increasing viscosity with this gum‐based thickening agent significantly improved airway protection mechanisms by reducing time to LVC. Increasing bolus viscosity also caused a slight, but significant, increase in oral residue and decreased tongue propulsion forces, and decreased bolus velocity at high viscosity levels without any significant effect on pharyngeal residue. Finally, the study shows that the gum‐based thickener is safe and well tolerated in patients with PSOD as reflected by the low number of AEs.

The chronic PSOD population is a phenotype of patients with OD that is growing in Europe, due to the increasing incidence of stroke events (from 1.1 million per year in 2000 to an estimated 1.5 million per year in 2025,^30^ the progressive increase in the prevalence of stroke survivors, and the high prevalence of OD among these patients (50%‐81%),^5^ even among those with mild strokes (45%).^3^ We and others have found that mild stroke survivors are at high risk of malnutrition 3, 31 and that aspiration pneumonia is the main cause of 1‐year mortality among them.^6^ The main result of our study is the viscosity‐dependent effect on safety of swallow with this xanthan gum‐based thickening agent in these patients with PSOD allowing safe deglutition in almost all these poststroke survivors with OD. The therapeutic range of this thickening agent in this phenotype of patients is 150‐800 mPa s, as 150 mPa s was the lowest viscosity to have a significant effect on the safety of swallowing and 800, 1400, and 2000 mPa s showed a similar level of protection. Aspiration is the most severe impairment in swallowing safety. For this parameter, the minimal viscosity with a significant effect was 250 mPa s, which suggests a therapeutic range starting at 250 mPa s. However, current results on aspiration can be considered inconclusive to establish the lower level of the therapeutic range because the study was not powered for this parameter. Low sample size, which was partly driven by the safety rule, might have prevented us from finding significant effects on aspiration at the lowest tested viscosity, that is, 150 mPa s., which was proven effective with regard to safe swallowing. For the main viscosities tested, significant differences in the therapeutic effect on safety of swallow vs liquid were found, and increasing bolus viscosity above 800 mPa s did not cause any further significant increase in the safety of swallow in this phenotype of patients. As far as we know, this is the first study to assess the effect of seven different viscosities in patients with PSOD. Our results suggest that, using this specific thickening agent, healthcare providers can cover the therapeutic needs of this phenotype of dysphagic patients by using a viscosity between 150 and 800 mPa s.

A major question that arises from these results is how to prescribe the optimal viscosity level of this thickening agent to PSOD. Firstly, these products should be labeled appropriately to promote their safe use.^32^ Secondly, accurate clinical methods should be used to diagnose OD and to prescribe which viscosity is the most appropriate for each patient with PSOD, as not all these patients can be assessed by instrumental exploration.^34^ Multiple consistency methods for clinical diagnosis of poststroke OD—such as GUSS and the V‐VST—have been recently recommended in PSOD in a guideline developed by the ESO and the ESSD and can be adapted to these viscosities.^33^ For the V‐VST—that uses only two levels of thickened viscosity—250 and 800 mPa s can be considered as the most appropriate; 800 mPa s as the viscosity providing the maximal significant therapeutic effect for this thickener; and 250 mPa s for patients with less severe safety impairment as a safe and comfortable intermediate value providing a significant therapeutic effect vs thin liquid and vs 800 mPa s.^22^, ^24^

Thickeners are widely used in poststroke OD as a compensatory therapeutic strategy to avoid aspiration. In a previous study on similar patients with PSOD, it was found that thickening liquids with either modified starch (MS) or xanthan gum‐based (XG) thickeners had a strong therapeutic effect on safety of swallow.^20^ The prevalence of safe swallow using MS and XG thickeners increased with bolus viscosity reaching up to 89%‐92% of patients with PSOD at higher viscosity levels (4000 mPa s for MS and 1700 mPa s for XG), above those used in the present study. In this previous study, the MS thickener strongly increased pharyngeal residues, whereas the XG increased oral residue at 1700 mPa s but did not increase pharyngeal residue at any viscosity. Timing of airway protection mechanisms (LVC) and bolus velocity were not affected by either of the thickener agents.^20^ This was one of the first studies showing an advantage for XG thickeners over MS in PSOD, due to its strong therapeutic effect on safety, low pharyngeal residue, and amylase resistance. The present study is a step forward as the therapeutic effect on safety of swallow is also very high (92.1% for 800 mPa s) and is achieved at lower viscosity levels, the absence of pharyngeal residue is similar, Nutilis Clear^®^ is unaffected by amylase, and—a new finding—increasing viscosity with this thickener causes a significant reduction of time to LVC over thin liquid. Videofluoroscopic studies have shown that the time to LVC is a critical event in the occurrence of penetrations and aspirations, causing unsafe deglutition, and time to LVC ≥340 ms predicts unsafe swallow in chronic PSOD patients.^20^ Such a delay in time to LVC in PSOD associated with impaired safety of swallow was also observed in this study, almost doubling the time to LVC of healthy people,^4^ and was slightly above that previously described in comparable patients.^12^ Reduced pharyngeal sensitivity and impaired conduction and cortical integration of pharyngeal sensory inputs at the stroke site is a key feature of chronic PSOD and has been closely associated with impaired safety of swallow and delayed time to LVC.^13^ In fact, sensory feedback from the bolus is critical to tailor the motor component of the swallow response. Therefore, the reduction in time to LVC caused by the thickening agent suggests a mode of action beyond a simple “compensatory” effect.^12^, ^13^ Another relevant result of the study is that increasing viscosity—which is a measure of the fluid resistance to bolus flow—reduces bolus propulsion force and bolus velocity at viscosities greater than 450 mPa s. This effect might explain the slight, but significant, increase in oral residue as tongue strength is reduced in these patients.^12^ This result agrees with a previous study from our group which concluded that impaired safety of swallow in chronic poststroke patients was caused by specific impairments in swallow response such as a delay in the airway protection mechanisms and weak tongue propulsion force.^12^ Those results led to a claim that treatments for these patients should be targeted to improve these critical biomechanical events (delay in LVC and reduce tongue strength). We recently studied the natural history of swallow function during the 3‐month period after stroke and found 26% of poststroke patients developed new signs/symptoms of ineffective swallow related to poor functional, nutritional, and health status and institutionalization.^35^ Another study on stroke patients concluded that tongue weakness was also caused by reduce muscle mass of swallow muscles and poststroke sarcopenia.^36^ Our present results of a reduced bolus propulsion force with the higher viscosities further suggest that stroke patients also need specific nutritional and rehabilitation procedures to increase bolus propulsion forces and tongue strength by fighting poststroke sarcopenia. Interestingly, pharyngeal residue, more related to pharyngeal clearance caused by pharyngeal constrictors, was unaffected by increasing viscosity.^35^

In the present study, increasing shear viscosity was obtained by adding increasing amounts (grams) of the gum‐based thickener to a mixture of water and contrast agent. The obtained shear viscosity is the independent variable for this study. Besides shear viscosity, other rheological proprieties such as elasticity, adhesiveness, and cohesiveness and different extensional viscoelastic behaviors also may play a role in swallow physiology.^18^ The assessment of the effect of extensional flows on viscosity of thickening agents is now under development, and the potential influence of these rheological properties on swallow safety and efficacy in patients with OD is still unknown.

Our study has some limitations. The first one arises from its experimental design as we included a pass/fail safety rule to protect patients from dangerous and unnecessary repeated aspirations. Due to our design, not all patients received all the viscosities, especially the lowest levels. This is a quite common situation in pharmacologic/physiologic studies, to minimize the possibility of serious AEs to patients, for example, during progressive effort tests.^27^ A similar “safety rule” was used in all our previous studies with thickening agents as requested by the Ethical Committee.^16^, ^25^ Because it is clinically relevant information, in‐between viscosity comparisons were performed by imputing the data of the missing values from the safety rule by carrying the last observation forward. As a consequence of the design of the study, care should be taken interpreting these results. However, this design and our interpretation is the safest from the patient's perspective. Another limitation is that the transversal design of the study does not provide information on longer term clinical outcome, for instance whether the observed improved safety of swallowing with the thickener agent results in fewer respiratory infections. Future longitudinal randomized clinical trials should be performed to confirm the translation of the strong therapeutic effect of the gum‐based thickener on swallowing safety into clinical outcomes including incidence of nutritional and respiratory complications.^3^ Nutritional support and oral care must also be included in these protocols.

In summary, this study shows that increasing bolus viscosity with Nutilis Clear® causes a strong viscosity‐dependent effect on safety of swallow in PSOD without increasing pharyngeal residue. Our study suggests that the therapeutic effect of the thickener might be caused by specific effects on oropharyngeal physiology (mainly time to LVC and bolus velocity). To optimize this strong therapeutic effect, clinicians must provide early diagnosis of PSOD and the prescription of the required appropriate viscosity by multiconsistency clinical and/or instrumental methods. This might be appropriate to reduce nutritional and respiratory complications and improve the prognosis of patients with PSOD. We believe these findings will have implications for current clinical practice. Our study clearly shows that the therapeutic effect of thickening agents depends on shear viscosity levels, with a therapeutic range of 150‐800 mPa s for this xanthan gum‐based thickener multiple consistency methods for clinical diagnosis, and management of poststroke OD can be adapted to this viscosity range for this specific phenotype of patients with OD. This information will improve clinical practice by providing the specific levels of viscosity to cover the therapeutic needs of this phenotype of dysphagic patients. Fluid thickening must be integrated into compensatory multimodal treatments, such as the minimal‐massive intervention 37 or neurorehabilitation approaches, aiming to restore swallow function.^38^

## CONFLICT OF INTEREST

Declaration of personal interests: Clavé has served as consultant and received research funding from Danone Nutricia Research. Guida, Ripken, and Lansink are employees of Danone Nutricia Research.

## AUTHOR CONTRIBUTION

Clavé is a submission's guarantor. Clavé, Rofes, Vilardell, and Lansink involved in study concept and design. Arreola, Rofes, Martín, Nascimento, Ortega, and Bolívar‐Prados involved in selection of patients. Rofes, Vilardell, and Bolívar‐Prados involved in acquisition of data. Clavé, Guida, Ripken, and Bolívar‐Prados involved in statistical planning and analysis. Clavé, Guida, Ripken, Lansink, and Bolívar‐Prados involved in analysis and interpretation of data. Clavé, Guida, and Bolívar‐Prados involved in drafting of the manuscript. Clavé and Bolívar‐Prados involved in revision of bibliography. All authors approved the final version of the manuscript, including the authorship list.
