# Supplementary figures and images for: Effect of a gum‐based thickener on the safety of swallowing in patients with poststroke oropharyngeal dysphagia
Source: Neurogastroenterol Motil. 2019 Aug 11;31(11):e13695. doi: 10.1111/nmo.13695 (PMC6852432; doi:10.1111/nmo.13695)

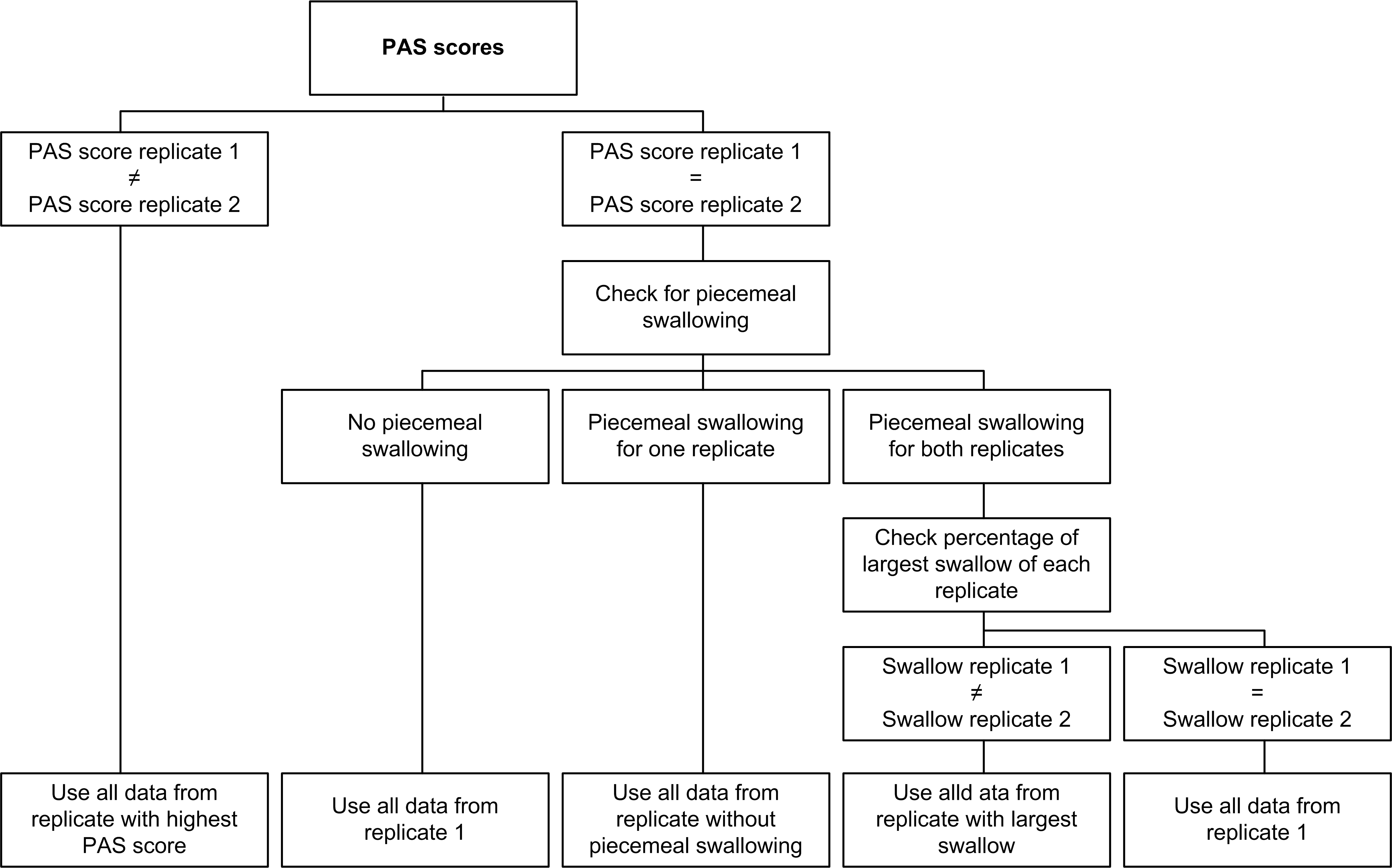

Supplement: Supplementary file 1 [file NMO-31-na-s001.tif]

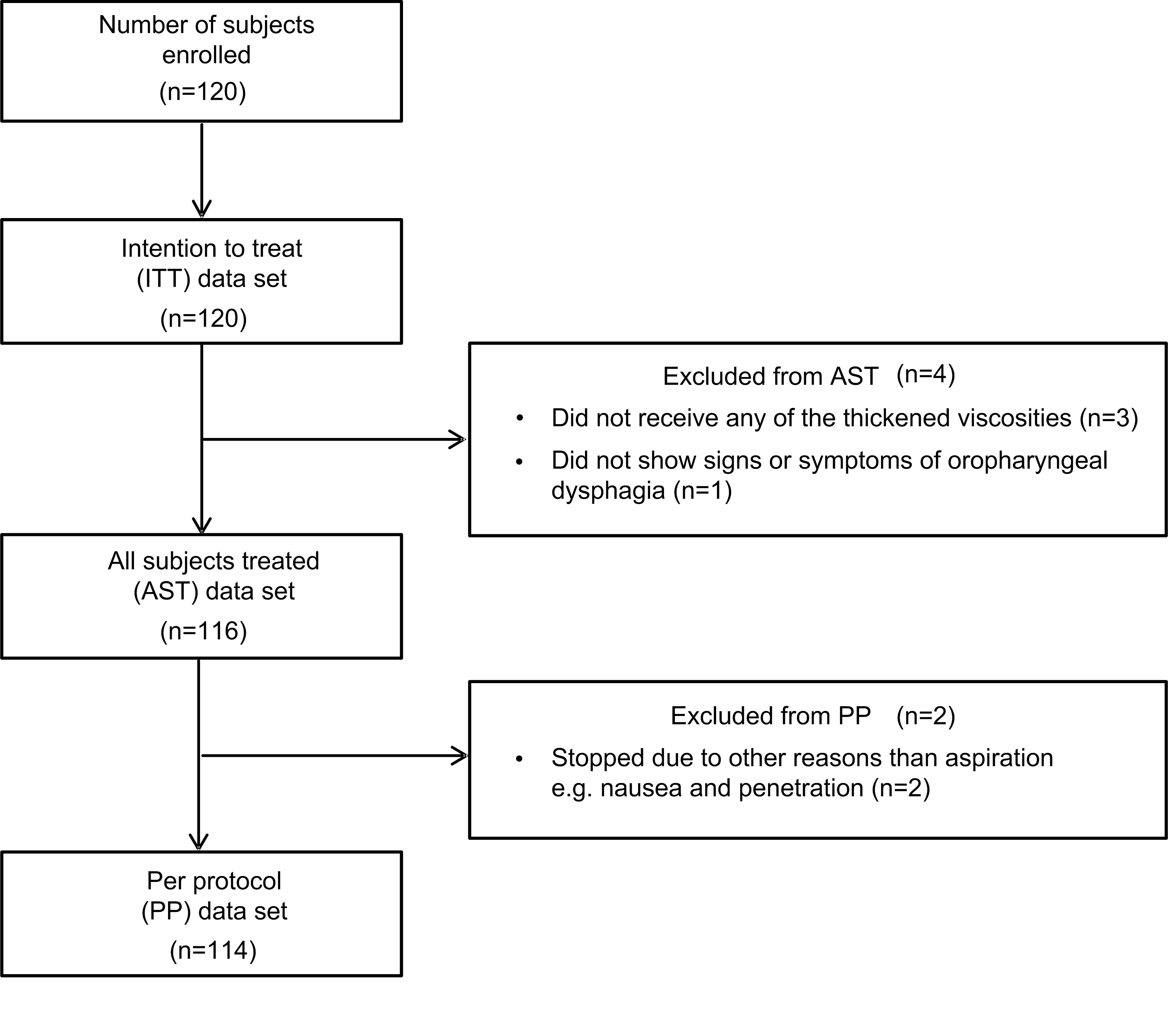

Supplement: Supplementary file 2 [file NMO-31-na-s002.tif]

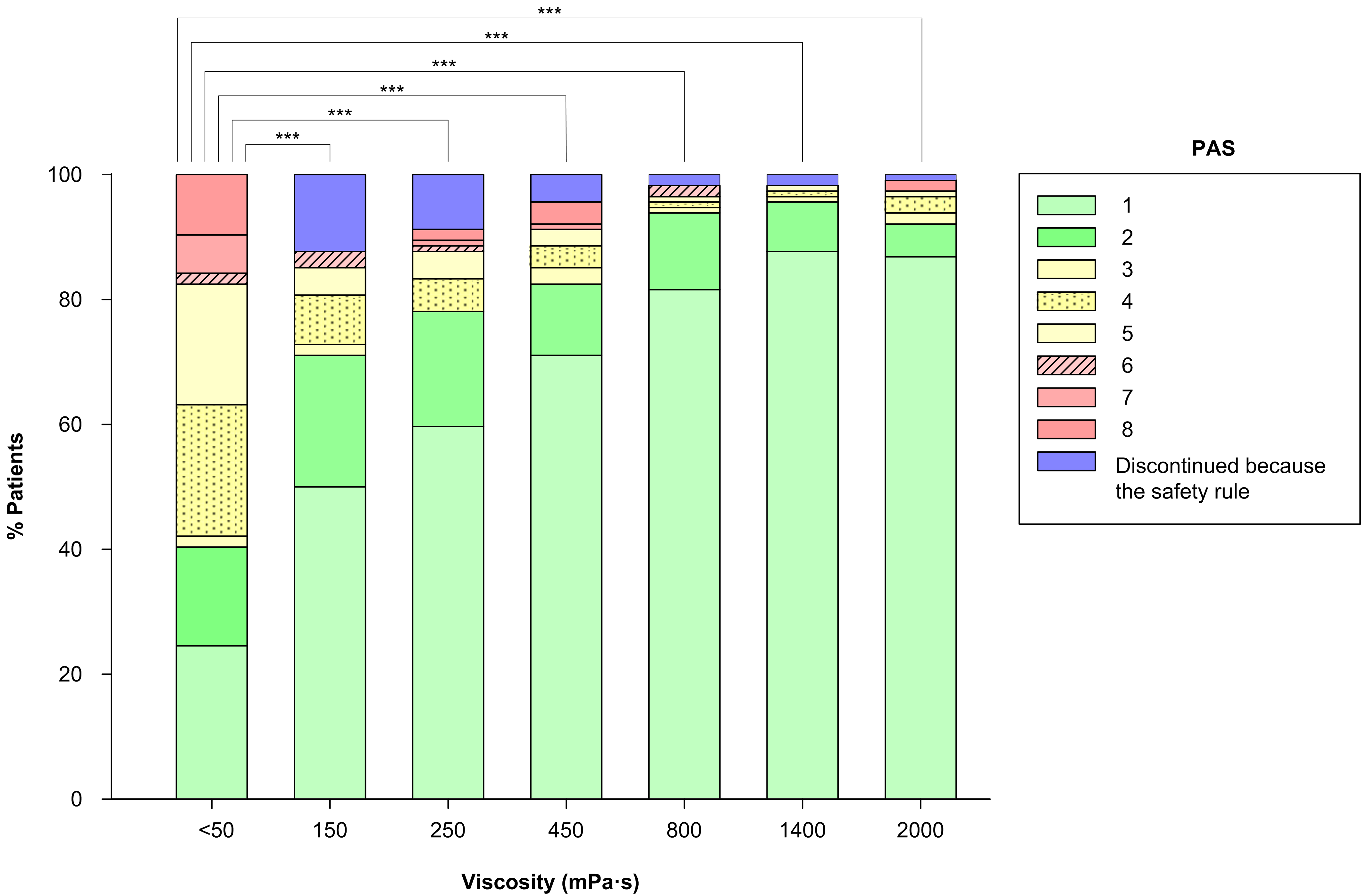

Supplement: Supplementary file 3 [file NMO-31-na-s003.tif]
