# Supplementary material for: Effect of a gum‐based thickener on the safety of swallowing in patients with poststroke oropharyngeal dysphagia
Source: Neurogastroenterol Motil. 2019 Aug 11;31(11):e13695. doi: 10.1111/nmo.13695 (PMC6852432; doi:10.1111/nmo.13695)
Supplement: Supplementary file 4 [file NMO-31-na-s004.docx]

**SUPPORTING INFORMATION**

**Supplementary Table 1.** Epidemiological and clinical characteristics of the population included in the study.

| **DEMOGRAPHICS** | |
| --- | --- |
| **Patients (N)** | 114 |
| **Sex (female; %)** | 45.6 |
| **Age (years; mean±SD)** | 76.7±8.9 |
| **Nutritional status (MNA-SF; %)** | |
| Malnourished (0-7) | 16.7 |
| At risk (8-11) | 37.7 |
| Well nourished (12-14) | 28.9 |
| Missing data | 16.7 |
| **Type of stroke (%)** | |
| Haemorrhagic | 11.4 |
| Ischemic | 78.1 |
| **Time after stroke (days; median Q1-Q3)** | 56 (41-170) |
| **NIHSS (mean±SD)** | |
| NIHSS in acute phase at admission | 7.5±6.8 |
| Missing data (N) | 23 |
| NIHSS in acute phase at discharge | 5.3±5.9 |
| Missing data (N) | 28 |
